# Finding Predictors of Leg Defects in Pigs Using CNV-GWAS

**DOI:** 10.3390/genes14112054

**Published:** 2023-11-08

**Authors:** Lyubov Getmantseva, Maria Kolosova, Kseniia Fede, Anna Korobeinikova, Anatoly Kolosov, Elena Romanets, Faridun Bakoev, Timofey Romanets, Vladimir Yudin, Anton Keskinov, Siroj Bakoev

**Affiliations:** 1 Federal State Budgetary Educational Institution of Higher Education, Don State Agrarian University, 346493 Persianovsky, Russia; lgetmantseva@cspfmba.ru (L.G.); kolosov777@gmail.com (A.K.);; 2 Federal State Budgetary Institution, “Center for Strategic Planning and Management of Medical and Biological Health Risks” of the Federal Medical and Biological Agency, 10/1 Pogodinskaya St., 119121 Moscow, Russia; kfede@cspfmba.ru (K.F.); akorobeinikova@cspfmba.ru (A.K.);

**Keywords:** pig, SNP, CNV, GWAS, leg defects

## Abstract

One of the most important areas of modern genome research is the search for meaningful relationships between genetic variants and phenotypes. In the livestock field, there has been research demonstrating the influence of copy number variants (CNVs) on phenotypic variation. Despite the wide range in the number and size of detected CNVs, a significant proportion differ between breeds and their functional effects are underestimated in the pig industry. In this work, we focused on the problem of leg defects in pigs (lumps/growths in the area of the hock joint on the hind legs) and focused on searching for molecular genetic predictors associated with this trait for the selection of breeding stock. The study was conducted on Large White pigs using three CNV calling tools (PennCNV, QuantiSNP and R-GADA) and the CNVRanger association analysis tool (CNV-GWAS). As a result, the analysis identified three candidate CNVRs associated with the formation of limb defects. Subsequent functional analysis suggested that all identified CNVs may act as potential predictors of the hock joint phenotype of pigs. It should be noted that the results obtained indicate that all significant regions are localized in genes (CTH, SRSF11, MAN1A1 and LPIN1) responsible for the metabolism of amino acids, fatty acids, glycerolipids and glycerophospholipids, thereby related to the immune response, liver functions, content intramuscular fat and animal fatness. These results are consistent with previously published studies, according to which a predisposition to the formation of leg defects can be realized through genetic variants associated with the functions of the liver, kidneys and hematological characteristics.

## 1. Introduction

One of the most complicated aspects of modern genome research is determining the relationship between genetic variability and phenotypes. In studies of the genetic architecture of breeding significant traits of farm animals, the main accent is focused on the analysis of SNPs (single nucleotide polymorphism). Moreover, structural variants, some of which are copy number variants (CNVs), have recently become of keen interest. A CNV is usually defined as a region of DNA approximately 1000 bp in size consisting of inversions, balanced translocations or genomic imbalances (insertions and deletions). Compared to SNPs, CNVs span wider chromosomal regions and could potentially be responsible for changes in gene structure, modifications in regulation and lead to significant phenotypic effects.

CNVs can affect gene expression as well as regulation, with potentially large phenotypic effects. Several human studies of CNVs have shown an association with Mendelian diseases and complex genetic disorders, such as schizophrenia [1], cancer [2,3] and various birth defects [4]. Similarly, in livestock, more and more studies are proving that CNVs have causal effects on phenotypic variation, for instance, CNVs in intron 1 of SOX5, causing the pea scallop phenotype in chickens [5], intronic duplication of 4.6 bp in STX17 is associated with hair graying and melanoma in horses [6], duplication of FGF3, FGF4, FGF19 and ORAOV1 leads to comb hair and predisposition to dermoid sinus cysts in Ridgeback dogs [7] and CNV and missense mutations of the agouti signaling protein (ASIP) gene lead to different coat coloration in goats [8]. However, at the current time, this topic has not been sufficiently studied in pigs. KIT is the first pig gene for which a gene duplication and splice mutation leading to exon 17 skipping have been proven to be responsible for the dominant white phenotype and peripheral blood cells (erythrocyte numbers and measures, total and differential leucocyte numbers, haematocrit and haemoglobin levels and serum components) [9,10,11]. CNVs have been associated with several phenotypes in pigs, such as coat color [12], stud thickness (BF) [13,14] and meat quality [15], demonstrating that CNVs can be considered promising markers of economically important traits.

Various methods have been used to detect CNVs, ranging from robust cytogenetic approaches such as karyotyping and fluorescence in situ hybridization to genome-wide in silico CNV prediction. Recently, significant improvements have been made in the accuracy and performance of CNV identification. The use of microarrays and next-generation sequencing technologies allows for a comparison of CNVs among populations on a genome-wide scale. In addition, sequencing methods open up the possibility of identifying CNVs in complex regions of the genome [16]. To detect CNV signatures in farm animals, including pigs, the most common approach is SNP chip data-based analysis [17]. As a result, CNV prediction software packages, such as PennCNV [18], QuantiSNP [19], GADA [20] and others, have been developed and published.

In pigs, as in many other species, insufficient knowledge is still apparent regarding the properties of CNVs. Of all the topics related to CNVs, knowledge of their functional effects is the most limited. Despite the wide range in the number and size of CNVs reported in previous studies, results have shown that a large proportion of CNVs are probably shared between different breeds, and their functional effects are underestimated in breeding pigs.

To date, in the field of livestock genomics, most studies have only focused on CNV detection, and few studies have been aimed at detecting possible phenotypic CNVs in livestock using genome-wide association studies (GWAS) [17]. Thus, in a study by Wang et al. [15], a GWAS was conducted between CNVs and meat quality traits in pigs. After false discovery rate correction, a total of eight CNVs were identified on six chromosomes that were significantly associated with at least one meat quality trait. Qiu et al. [21] identified 35 CNVRs with significant associations with growth and body condition traits using CNVR-based GWAS. Ten of these CNVRs were associated with both ADG and AGE traits in Duroc pigs, and four CNVRs showed significant associations with ADG, AGE and BFT, which may be considered a pleiotropic role for CNVR in regulating pig growth and fat deposition. According to the results of Ding et al. [22], association analysis identified 10 CNVRs, one of which was associated with the loin muscle area, one with loin muscle depth, and eight with lean meat percentage (LMP) in Duroc pigs.

One of the major problems faced by pig producers is the spread of limb defects, including bumps and growths in the area of the hock joint on the hind legs [23]. The identification of genome regions directly or indirectly associated with limb conditions can help to identify genetic variants and use them as genetic markers for the selection of breeding pigs. In this regard, it will be useful to integrate copy number variability (CNV) analysis to improve knowledge of the genetic architecture of complex traits such as limb defects in pigs.

## 2. Materials and Methods

### 2.1. Sample for Analysis

The studies were conducted on pigs of the Large White breed (*n* = 100) under the same conditions of housing and feeding. The presence of bumps/narrows in the area of the hock joint on the hind legs was determined by visual inspection in animals when they reached 100–110 kg. Pigs were divided into two groups based on leg conditions: presence/absence (*n* = 45/*n* = 55, respectively) of bumps/narrows. Genomic DNA was isolated from tissue (ear plucking) using a DNA-Extran-2 reagent kit (Syntol Ltd., Moscow, Russia). GeneSeek^®^ GGP Porcine HD Genomic Profiler v1 was used for genotyping.

### 2.2. Calling CNV

Three programs were used to identify CNVs from SNP array data: PennCNV v.1.0.4 [18], QuantiSNP v2.3 [19] and R-GADA 2.0.1 [20]. PennCNV and QuantiSNP use a Hidden Markov Model (HMM), while GADA uses a Sparse Bayesian Learning (SBL) model.

PennCNV uses the SNP marker information in a text file format. The Illumina microarray report file was reformatted using split_illumina_report.pl. False positives were removed with filter_cnv.pl, allowing the CNV to be selected with a standard deviation LRR of less than 0.3. QunatiSNP requires the original Illumina report file as input files. The results with a logarithmic coefficient below 10 were removed, as recommended by the creators of the program. The R GADA package was taken as the third program to call CNV. The first step was to find the most likely candidate points for CNV using the Sparse Bayesian Learning (SBL) model function.

For the further analysis of CNVs, it is often necessary to combine individual CNVs into regions. For this purpose, a Reciprocal Overlap (RO) procedure was performed using the R CNVRanger package v.1.18.0 [24], which combines individual CNVs into regions using the specified overlap threshold ro.thresh. To convert individual CNVs into regions, the output files from the three methods PennCNV, QuantiSNP and GADA were combined into a single file. All CNVs longer than 5 Mb were removed, as CNVs of this length are likely to be false positives. Another criterion for selecting CNVs for inclusion in the regions is the presence of overlapping events between the two algorithms. This step was performed using the subsetByOverlaps function of the R GenomicRanges package. The data was converted into a file containing the chromosome number, the beginning and end of the CNV, the name of the sample and the state of the CN. It was then converted using GRangesList into a format suitable for CNV data analysis. This format displays a list of samples, each of which in turn has its own set of CNVs. Then, the function populationRanges was used with the parameters mode = “RO” and ro.thresh = 0.51, which denote the Reciprocal Overlap method used and the overlap threshold between CNVs in shares.

### 2.3. Annotation of Quantitative Trait Loci Located in CNVs Regions

The R GALLO package v. 1.3 [25] was used to analyze the effect of CNV regions on quantitative trait loci. This package offers functionality for annotation and visual presentation of results. This analysis required a gff file from the AnimalQTL database, which contains the positions of the quantitative trait loci of the pig. The results of merging individual CNVs into regions were downloaded in text format. The package’s find_genes_qtls_around_markers function was used to annotate the CNV at the quantitative trait loci. The graph corresponding to the percentage of traits from the most severely affected CNVRs was constructed based on the annotated data and the function plot_qtl_info. The most enriched features affected by CNVR in all QTL classes were visualized using the QTLenrich_plot function. Also, based on CNVR annotation data, charts were created reflecting external traits, meat traits, health-related traits and reproductive traits. The above graphs were plotted using plot_qtl_info with the class of the corresponding feature indicated.

### 2.4. Association Analysis of CNV with Phenotype

CNV-GWAS analysis was performed to identify the association of CNV with defects in legs, which was phenotypically present in 45 of the 100 pigs studied. The CNVRanger package provides the function to implement association analysis developed for CNV calls. Since CNVs from SNP arrays are constructed from probes that represent common polymorphisms with allele frequencies greater than 1%, analyses based on standard logistic regression were performed. For GWAS, the *p*-value of the probe that is part of the CNV was selected. The analysis requires the chromosome number, the starting and ending positions of the CNV, the sample names and the integer state of the copy number. CNV calls were provided in GRangesList format, which allows for the separation of CNVs by pattern. After converting the CNVs text file, information about the phenotypes of the studied animals was read. The information was provided as a text file that contained the name of the sample and information about the phenotypic manifestation of presence/absence bumps/narrows in the area of the hind limbs of pigs: (as 0–absence (healthy) and 1–presence (sick)). The analysis also required the SNP map file, which contained information about the chromosome number, position and polymorphism identifier. Immediately prior to GWAS, all files were organized and configured using the setupCnvGWAS function. The CNV-GWAS analysis using the cnvGWAS function resulted in a minimum *p*-value of the probe included in the CNV. Multiple test corrections were performed based on the FDR test built into the CNVRanger package used. The results were visualized using a Manhattan plot.

## 3. Results and Discussion

**Figure 1:**
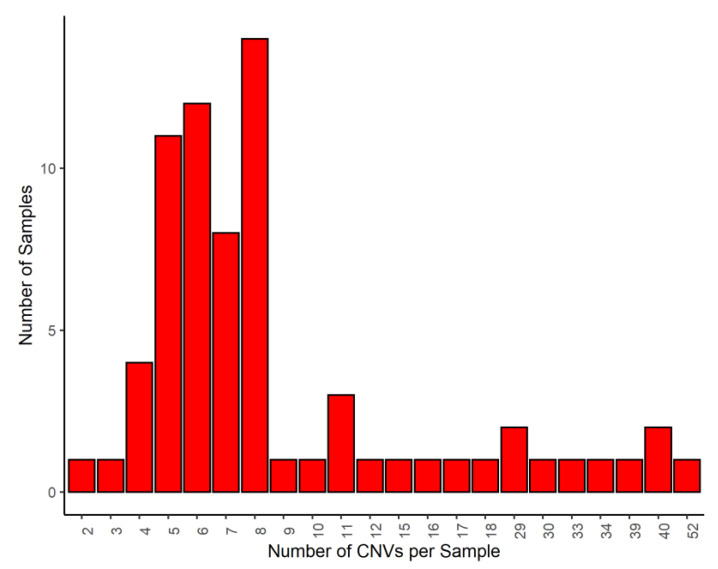
Distribution of the amount of CNVs per sample.

Three algorithms (PennCNV, QuantiSNP and GADA) were used to identify 3098 CNVs: 429, 810 and 1859, respectively. CNVs less than 5 Mb were excluded and only CNVs identified by at least two algorithms were selected for analysis. As a result of the studies, 775 CNVs were used for further analysis. The length of deletions ranged from 66 bp to 992 bp. The length of duplicates ranged from 28.9 to 4.9 Mb. On average, eight CNVs were localized in the genome of one animal. The maximum and minimum number of CNVs per animal in the study sample was fifty-two and two CNVs, respectively (Figure 1).

**Figure 2:**
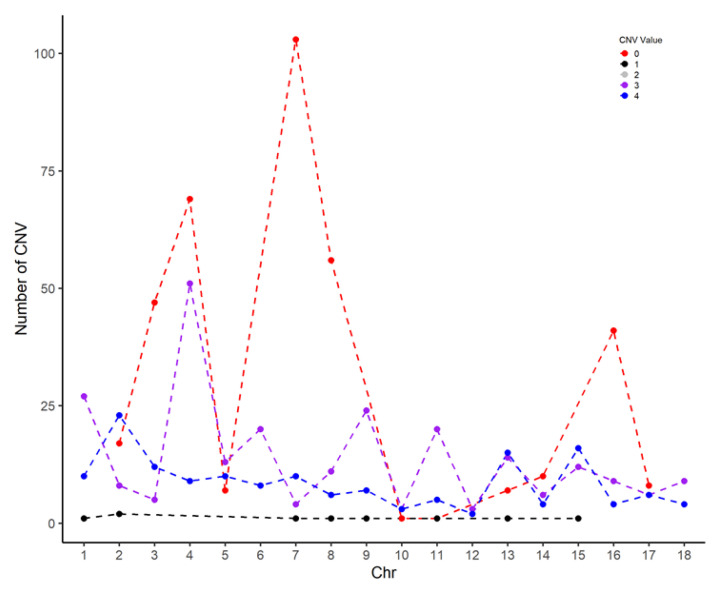
Distribution of CNVs across chromosomes.

Figure 2 shows the distribution of different CNVs across chromosomes. As can be seen, duplications are evenly spread across chromosomes, while deletions are mainly localized on chromosomes 3, 4, 7 and 8.

(Interpretation of CNV values: 0–complete deletion; 1–single copy deletion; 2–normal (heterozygote) and normal (homozygote); 3–single duplication; 4–double duplication).

**Figure 3:**
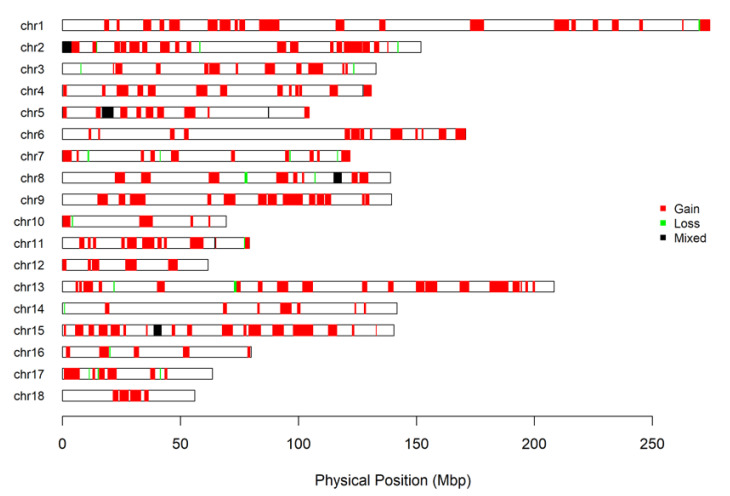
Genome-wide map of CNVR in Large White pigs.

Combining CNVRs into regions produced 246 CNVRs, of which 112 CNVRs occurred in two or more pigs (Add file 2). The size of these regions in the sample studied ranged from 67 bp to 8.2 Mb. Twelve regions contained copy losses, ninety-one regions contained copy gains and nine regions included both types (losses and gains) of copies (Figure 3).

**Figure 4:**
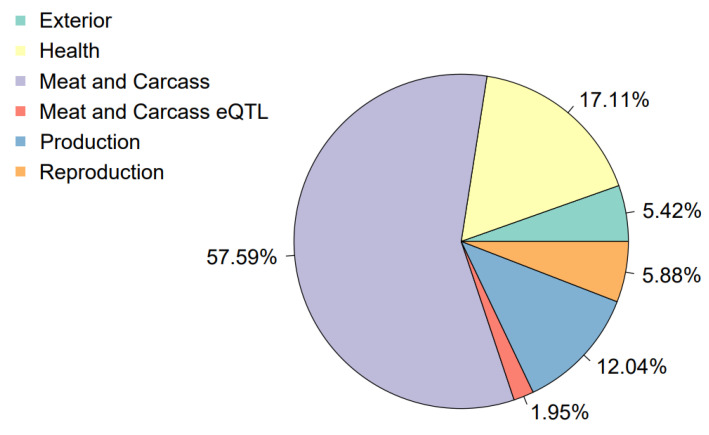
Percentage of QTLs affected by CNVRs.

According to the CNVR annotation of quantitative trait loci and the visualization of results using R package GALLO, it was found that most of the regions (about 57.59%) affected loci associated with traits responsible for meat qualities and the carcass of the pig. In addition, 17.04% QTLs were associated with health and 12.04% with the production cycle. The lowest number of CNVRs overlapped with loci characteristic of reproductive traits and exteriors (Figure 4).

Considering each trait class in more detail, in the exterior class (Supplementary Materials File S1, Figure S1), the most frequent CNVRs are found at loci responsible for coping behavior, the osteochondrosis score, ear weight and iris pigmentation. Coping behavior traits are associated with coping styles for coping with stressful situations. Styles differ in the level of aggression, which depends on the neuroendocrine differences of individuals [26]. The effect of CNVR on the osteochondrosis score is also large in terms of percentage and significance for the individual pig. Osteochondrosis disease is a common disease and is a major cause of leg weakness in pigs [27].

Through imaging QTL percentages affecting the health (Supplementary Materials File S1, Figure S2) of CNVR-affected individuals, a high percentage of loci associated with the viral load of porcine reproductive and respiratory virus was observed. This virus is dangerous to health because it causes pneumonia in growing pigs and abortion in sows. Enrichment of this CNVR region is probably related to the immune response to the PRRS virus. This locus overlapped our detected regions 4:127341468–127373937 and 4:127379776–127379842, which occurred in 32 pigs as copy gain and 69 as copy loss, respectively. Loci affecting the percentage of CD8-positive leukocytes and CD3, CD8-negative leukocyte percentages were also significantly affected. CD8+ leukocytes, or cytotoxic T lymphocytes of the CD8+ phenotype, are produced in the thymus and express a T-cell receptor (TCR) that reacts to foreign antigens. These cells are an essential component of the adaptive immune system and play an important role in protecting against viruses, bacteria and tumors.

Among the reproductive traits, the nipple number, pubertal age and traits responsible for the amount of corpus luteum were more enriched in CNVR (Supplementary Materials File S1, Figure S3). The CNVR annotation of quantitative trait loci and visualization of the results using the R GALLO package revealed that the majority of regions, about 57.59%, affected loci associated with traits responsible for the properties of meat and the carcass of the pig. A large part of the CNVR affects the QTLs responsible for the average meat gain per day and feed conversion; in other words, the efficiency of fattening animals (Figure S4). The results of the enrichment of QTLs by variations visualized by GALLO showed significant enrichment in loci responsible for aspartate aminotransferase activity, pH for the dorsal long muscle and the average backfat thickness. Basically, all of the most enriched loci are represented by a class of traits associated with meat quality, reproductive properties and productive qualities. The most significant events are reflected on the bubble diagram as circles in the darkest color and are considered significant based on the greater extent of the CNVR region. Quantitative traits are referred to as polygenic effects; that is, they are the product of two or more genes. Many economically useful traits are inherited by a complex polygenic type and are controlled by many genes located in the QTL loci in agricultural animals. The analysis of overlapping QTLs by copy number variations showed that many of the traits affected are responsible for productive traits. These results are consistent with the previous analysis, since many genes affected by CNV are important for lipid metabolism and adipocyte formation. Information on the affected loci of quantitative traits can be used to study the impact of CNV on these traits. If positive or negative manifestations are detected as a result of animal transcriptome analysis, it is possible to isolate the most useful markers for meat production and subsequent selection.

**Figure 5:**
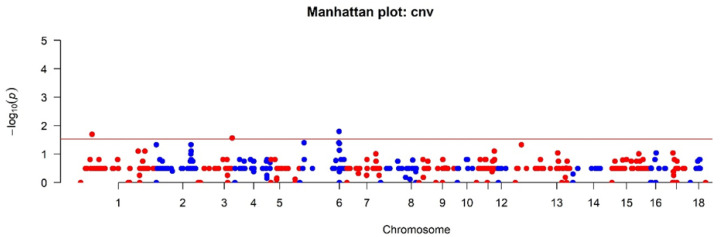
Manhattan plot for CNVR, red: odd chromosomes; blue: even chromosomes.

The association of CNVs with the phenotype was analyzed to confirm or refute the hypothesis that these CNVs influence defects in the legs of the pigs studied. Multiple testing corrections were performed based on the FDR test built into the CNVRanger package used. In total, we found three CNVR groups (*p*  <  0.05 after false discovery rate correction) with the studied phenotype. The SNPs found: 6:142358005 *p*-value = 0.01593, 1:41300464 *p*-value = 0.0201, 3:123404335 *p*-value = 0.02714 are localized to the following CNVRs: 6:142358005-143865882 gain type, 1:41300464–42881624 gain type, 3:123404335–123460528 gain type (Figure 5).

CNVR 6:142358005–143865882 encompasses two genes, CTH and SRSF11. The CTH gene encodes cystathionine γ-lyase, an enzyme involved in the biosynthesis of cysteine via cystathionine. Mutations in this gene contribute to impaired amino acid metabolism. The SRSF11 gene encodes a pre-mRNA splicing factor and is an arginine-serine-rich protein of the SR family [28].

The STRING database [29] was used to evaluate protein associations and to make suggestions about their functional associations. The main functional pathways for CTH are metabolic pathways (Metabolic pathways) (Supplementary Materials File S1, Table S1), which result in the formation or alteration required for the proper functioning of a biological system through the regulation of sulfur-containing amino acids (SAA), such as methionine (Met) and cysteine (Cys). Methionine is metabolized in the liver and small intestine by transmethylation, remethylation and transsulfuration. Cystathionine γ-lyase, encoded by the CTH gene, catalyzes the last stage of transsulfuration from Met to Cys [30].

In addition to protein synthesis, Met and Cys affect the production of cytokines, glutathione and amylase precursor proteins (APP). Insufficient amounts of these acids lead to a decreased immune response, antibody production and T-cell proliferation in pigs, as well as the development of lymphoid organs. All of these may be causes of defects in the limbs of pigs. The relationship between the SAA ratio and CTH gene expression suggests that CNVRs in this gene play a role in the metabolism of sulfur-containing amino acids, thereby provoking processes associated with cone/narrowleaf formation in pigs.

In addition to CTH, CNVR: 6:142358005–143865882 overlaps a region of the SRSF11 gene. CNV affecting this protein may influence the process of adipogenesis, as analysis of the SRSF11 transcriptome in Large White pigs has shown that this protein is hypothesized to be involved in adipocyte differentiation in muscle during the cell cycle [14]. Furthermore, CNVR 6:142358005–143865882 overlaps with QTL for traits from meat and carcass classes responsible for intramuscular fat content. Adipocyte hypertrophy occurs as a result of a metabolic shift toward lipogenesis [31]. Metabolic processes occurring in intramuscular fat adipocytes are associated with a risk of cardiovascular disease, insulin resistance and muscle tissue development [14].

The association of fatness markers with limb defects in pigs has also been reported in a study by Le et al. [32]. The researchers analyzed three breeds to identify genetic markers affecting lameness and leg weakness. Among the six most significant SNPs found in the Landrace breed was a variant in the SRSF12 gene. The result of this analysis showed the association of CNVR 6:142358005–143865882 with the studied limb defect phenotype.

The next most significant CNVR 1:41300464–42881624 partially affects the MAN1A1 gene, which encodes a protein involved in protein glycosylation. Currently, there are limited data on the associations of MAN1A1 with breeding traits in pigs. MAN1A1 has been determined to be involved in pathways of various types of N-glycan biosynthesis (various types of N-glycan biosynthesis and N-Glycan biosynthesis) (Supplementary Materials File S1, Table S2). Through glycosylation, cellular functions, including cell adhesion, are modulated [33] and proteins that play key roles in homeostatic functions, such as inflammation, morphogenesis and immunity, are modified [34].

Defects in the N-glycosylation of proteins are the most common causes of genetically determined diseases in which the synthesis of glycans, their attachment to glycoproteins and glycolipids and the synthesis of glycosylphosphatidylinositol are impaired [35]. Liver damage is a hallmark of almost all glycosylation disorders. The disease may manifest with hepatomegaly, cholestasis or liver failure. Severe psychomotor disturbances, hypotonia, seizures, craniofacial dysmorphism and gastrointestinal disorders are not uncommon. In the case of leg defects in pigs, it can be suggested that if the bumps on the limbs have any association with liver disease, CNVs in the MAN1A1 gene play a role in this process and can be considered markers of predisposition.

CNVR 3:123404335–123460528 is localized in the intergenic region and does not encompass any genes. However, today, it is becoming increasingly clear that mechanisms that play a significant role in the regulation of gene expression are localized in intergenic regions. In this regard, nearby genes from CNVR 3:123404335–123460528 were examined. In the context of the study, the gene of interest was LPIN1 (3: 124940548–125074123), which belongs to the LPIN family of proteins. These are Mg2+-dependent phosphatidic acid phosphatase enzymes that perform a key reaction in glycerolipid biosynthesis [36]. LPIN1 was identified during the positional cloning of a mutant gene underlying lipodystrophy in mice with fatty liver dystrophy (fld) [3]. LPIN1 is expressed in skeletal muscle and plays a key role in muscle function [37].

In pigs, the major functional pathways in which LPIN1 is involved are glycerolipid metabolism, fat digestion and absorption, metabolic pathways, glycerophospholipid metabolism and the phospholipase D signaling pathway (Supplementary Materials File S1, Table S3).

Lipin performs as a transcription factor, changing the expression of genes involved in fatty acid oxidation and mitochondrial metabolism. This dual function makes lipin a unique protein, without the correct functioning of which it is impossible for adipocytes and hepatocytes to function. Studies have shown that LPIN1 is involved in milk fat synthesis in goats [38], cattle [39] and buffalo [40]. The disruption of gene expression causes developmental delays in insects [41] and an increased risk of chronic diseases in mammals [42]. Lipin deficiency in humans may underlie diseases such as atherosclerosis, colitis, cancerous tumors and liver disease [43]. In mouse liver, LPIN1 deficiency prevents normal lipid accumulation and induces the expression of key adipogenic genes. There is no publicly available data supporting the association of LPIN1 with any traits or diseases in pigs. However, its role in glycerolipid and glycerophospholipid metabolism, fat synthesis and the phospholipase D signaling pathway provides strong evidence to suggest that LPIN1 may be associated with limb defects in pigs.

Thus, the results obtained indicate that all significant CNVs are localized (or close to them) in genes responsible for the metabolism of amino acids, fatty acids, glycerolipids and glycerophospholipids, thus associated with immune response, liver function, intramuscular fat content and animal fatness. The results obtained are in agreement with earlier findings [16], according to which the predisposition to the formation of lumps and growths in the area of the hock joint of limbs can be realized through genetic variants associated with liver function, kidney function, hematological traits, and susceptibility to infections, as well as be associated with the composition of lipids and fatty acids.

## 4. Conclusions

The purpose of this study was to identify CNVs and to analyze the supposed impact of these structural variations on the genome and phenotype of pigs. This study analyzed genotyping data from the SNP arrays of 100 pigs. These Large White pigs were divided into two groups depending on the leg condition: presence/absence of bumps/narrows in the area of the hock joint on the hind legs. A total of 775 CNVs were identified using the results obtained. This was based on at least two algorithms (PennCNV, QuantiSNP and the R package of GADA). The resulting CNVs were combined into 246 CNV regions. The QTL overlap analysis by CNVR revealed affected loci of the Meat and Carcas class 57.59%, Helth 17.04%, Exterior 5.42%, Reproduction 5.88%, Production 12.04% and eQTLs Meat and Carcas 1.97%.

This study also used GWAS analysis to examine CNVs as candidates for leg defects in pigs. In summary, CNV-GWAS showed three CNVRs localized at positions: 6:142358005–143865882 (overlapping the CTH and SRSF11 genes), 1:41300464–42881624 (overlapping the MAN1A1 gene) and 3:123404335–123460528 (nearby the LPIN1 gene). Functional analysis of the genes suggested that all identified CNVs may act as potential markers of the presence of bumps/narrows in the area of the hock joint on the hind legs in pigs.

Copy number variation (CNV) is the basis for the type of genetic variation that causes phenotypic differences between pigs and can serve as an alternative single-nucleotide polymorphism (SNP) molecular marker for genome-wide association studies (GWAS). The results presented in our work can be used in breeding work for early diagnosis of the predisposition of pigs to leg defects. Future research will focus on examining genetic variants, including CNV, for associations with both limb defects and performance traits, as these desirable variants for performance traits may also be associated with various defects in pigs.

## Data Availability

Data are contained within the article and Supplementary Materials.

## Supplementary Materials

The following supporting information can be downloaded at: https://www.mdpi.com/article/10.3390/genes14112054/s1, Supplementary Materials File S1, Table S1. Functional pathways for the CTH and SRSF11 genes (according to STRING database); Table S2. Functional pathways for the MAN1A1 gene (according to the STRING database); Table S3. Functional pathways for the LPIN1 gene (according to STRING database); Figure S1. Percentage of exterior class traits affected by CNVR; Figure S2. Percentage of health class traits affected by CNVR; Figure S3. Percentage of reproduction class traits affected by CNVR; Figure S4. Percentage of production class traits affected by CNVR; Supplementary Materials File S2, CNVR across chromosomes.
