# Supplementary material for: Finding Predictors of Leg Defects in Pigs Using CNV-GWAS
Source: Genes (Basel). 2023 Nov 8;14(11):2054. doi: 10.3390/genes14112054 (PMC10671522; doi:10.3390/genes14112054)
Supplement: Supplementary file 1 [file genes-14-02054-s001.zip › Supplementary file 1.pdf]

**Supplementary file 1**

Table S1. Functional pathways for the CTH and SRSF11 genes (according to STRING database)

| | |
|---|---|
| Relevant datasets in Sus scrofa: | |
| annotated pathway (KEGG). Name: Metabolic pathways | *CTH, MPST, CDO1, TST , MTR , GCLC , ENSSSCG00000023370 , MTHFR* |
| annotated pathway (KEGG). Name: Metabolic pathways | *CTH, MPST, CDO1, TST, MTR, GCLC* |
| annotated pathway (KEGG). Name: Selenocompound metabolism | *CTH, MTR, ENSSSCG00000023370* |
| curated pathway / complex polyadenylated, capped mRNA:CBC:EJC:TREX:SRSF proteins | *SRSF11 , SRSF7 , SRSF3* |
| curated pathway / complex mRNA polyadenylation | *SRSF11, SRSF7, SRSF3* |
| curated pathway / complex 3' end cleaved, ligated exon containing complex | *SRSF11, SRSF7, SRSF3* |
| curated pathway / complex mRNA 3'-end processing | *SRSF11, SRSF7, SRSF3* |
| curated pathway / complex Cleavage of mRNA at the 3'-end | *SRSF11, SRSF7, SRSF3* |
| curated pathway / complex Spliced mRNA:CBC:EJC:TREX | *SRSF11, SRSF7, SRSF3* |
| curated pathway / complex RNA Polymerase II Transcription Termination | *SRSF11, SRSF7, SRSF3* |
| Relevant information transferred from other organisms: | |
| curated pathway / complex (biocyc) superpathway of methionine degradation | Mus musculus: *Cth, Cdo1, Mtr* |
| curated pathway / complex (reactome) capped mRNA:CBC:EJC:TREX (minus DDX39B) | Drosophila melanogaster: *Srp54, x16, Rbp1* |
| curated pathway / complex (reactome) polyadenylated, capped mRNA:CBC:EJC:TREX:SRSF proteins | Drosophila melanogaster: *Srp54, x16, Rbp1* |
| curated pathway / complex (reactome) capped mRNA:CBC:EJC:TREX (minus DDX39B) | Rattus norvegicus: *Srsf11, Srsf7, Srsf3* |

Table S2. Functional pathways for the MAN1A1 gene ((according to the STRING database)

| **Relevant datasets in Sus scrofa:** | |
|---|---|
| annotated pathway (KEGG)<br>*Name: Various types of N-glycan biosynthesis* | *MAN1A1, MAN1C1, MAN1A2, LOC780412* |
| annotated pathway (KEGG)<br>*Name: N-Glycan biosynthesis* | *MAN1A1, MAN1C1, MAN1A2, LOC780412* |
| annotated pathway (KEGG)<br>*Name: Metabolic pathways* | *MAN1A1, MAN1C1, MAN1A2, LOC780412* |
| annotated pathway (KEGG)<br>*Name: Protein processing in endoplasmic reticulum* | *MAN1A1, MAN1C1, MAN1A2* |
| **Relevant information transferred from other organisms:** | |
| curated pathway / complex (biocyc)<br>*protein <i>N</i>-glycosylation processing phase (mammalian)* | Homo sapiens: *MAN1A1, MAN1C1, MAN1A2, MAN1B1* |
| curated pathway / complex (reactome)<br>intra-Golgi cargo:GOLGA5 dimer:GOSR1 Homo sapiens | Homo sapiens: *MAN1A1, MAN1C1, MAN1A2* |
| curated pathway / complex (reactome)<br>*COG complex:CUX1 dimer:GOLGA5 dimer:STX5:PalmC-YKT6:BET1L:GOSR1:intra-Golgi retrograde cargo* | Homo sapiens: *MAN1A1, MAN1C1, MAN1A2* |
| curated pathway / complex (reactome)<br>*The COG complex and CUX1 and GOLGA5 dimers contribute to intra-Golgi vesicle tethering* | Homo sapiens: *MAN1A1, MAN1C1, MAN1A2* |

Table S3. Functional pathways for the LPIN1 gene (according to STRING database)

| | |
|---|---|
| **Relevant datasets in Sus scrofa:** | |
| annotated pathway (KEGG)<br>*Name: Glycerolipid metabolism* | *LPIN1, DGAT,*<br>*MOGAT2, AGPAT2, AGPAT1, PPAP2C* |
| annotated pathway (KEGG)<br>*Name: Fat digestion and absorption* | *DGAT, MOGAT2, AGPAT2, AGPAT1, PPAP2C* |
| annotated pathway (KEGG)<br>*Name: Metabolic pathways* | *LPIN1, DGAT, AGPAT2, AGPAT1, PPAP2C* |
| annotated pathway (KEGG)<br>*Name: Glycerophospholipid metabolism* | *LPIN1, AGPAT2, AGPAT1, PPAP2C* |
| annotated pathway (KEGG)<br>*Name: Phospholipase D signaling pathway* | *AGPAT2, AGPAT1, PPAP2C* |
| **Relevant information transferred from other organisms:** | |
| curated pathway / complex (biocyc)<br>triacylglycerol biosynthesis | Mus musculus: *Dgat1*, *Agpat2*, *Agpat1*, *Ppap2c* |
| curated pathway / complex (biocyc)<br>triacylglycerol biosynthesis | Homo sapiens: *DGAT1, AGPAT2,*<br>*AGPAT1,PPAP2A,, PPAP2C* |
| curated pathway / complex (reactome)<br>Specific granule membrane proteins | Bos taurus: *DGAT1, AGPAT2* |
| curated pathway / complex (reactome)<br>Specific granule membrane proteins | Bos taurus: *DGAT1, AGPAT2* |

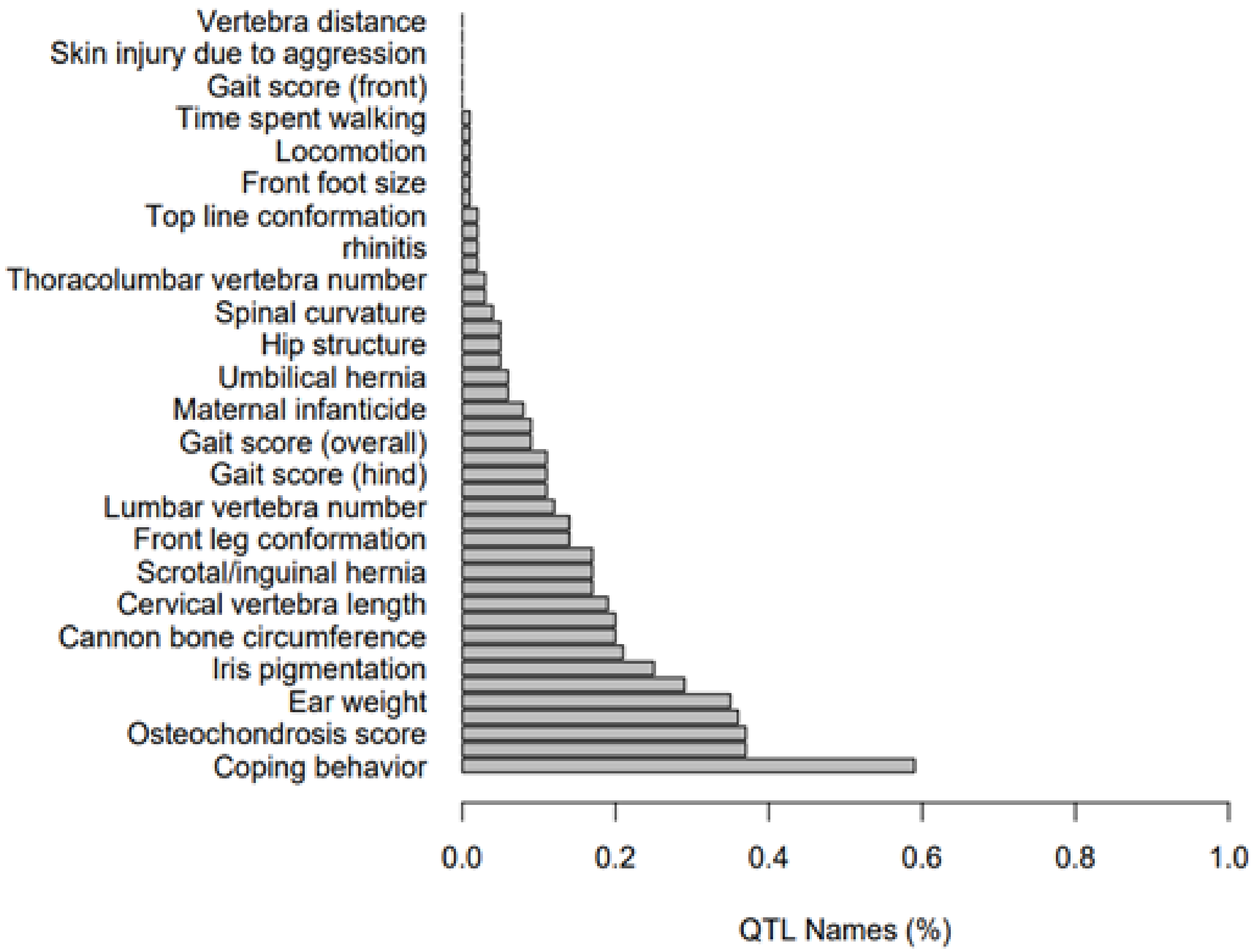


Figure S1. Percentage of exterior class traits affected by CNVR

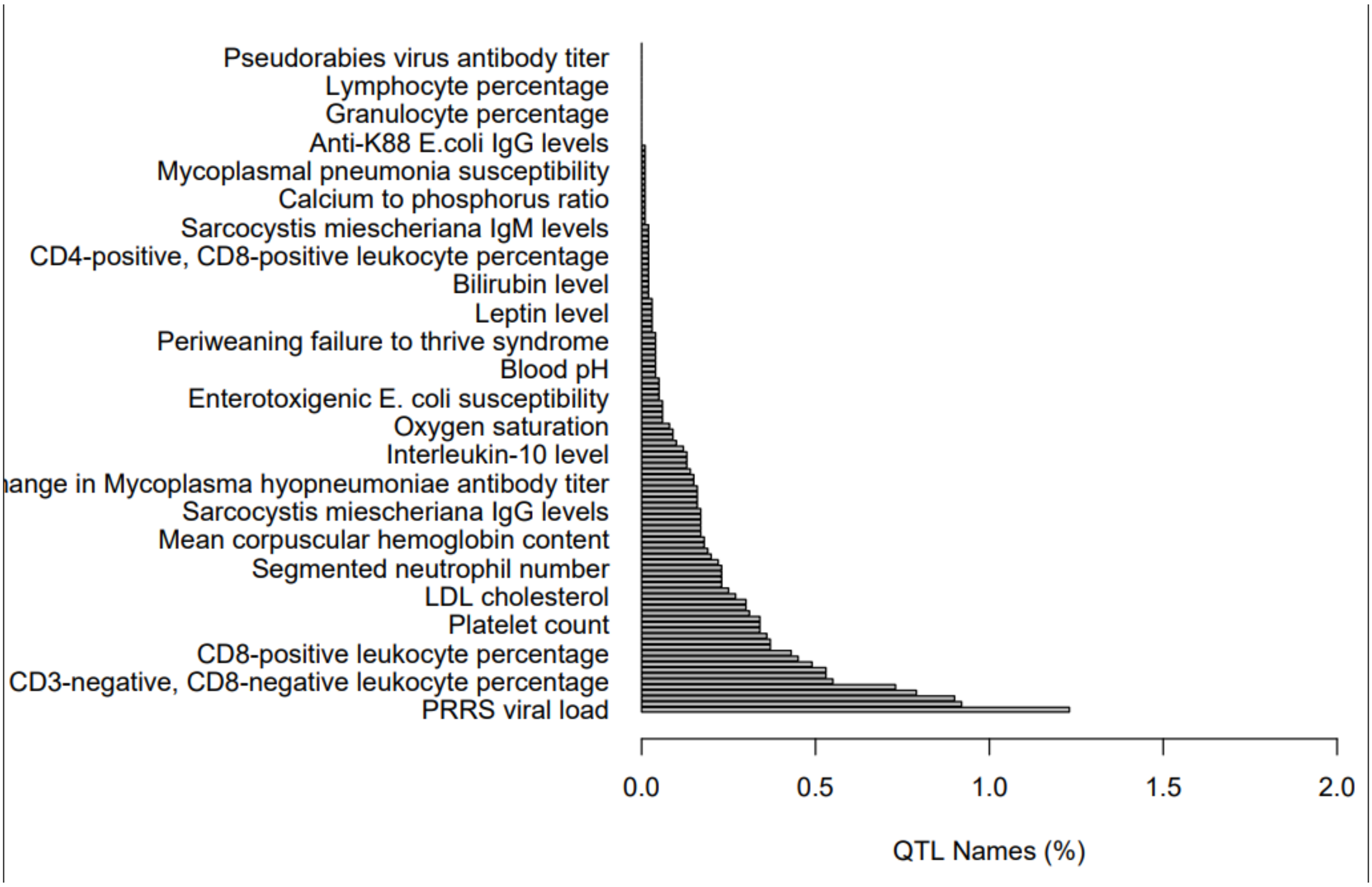


Figure S2. Percentage of health class traits affected by CNVR

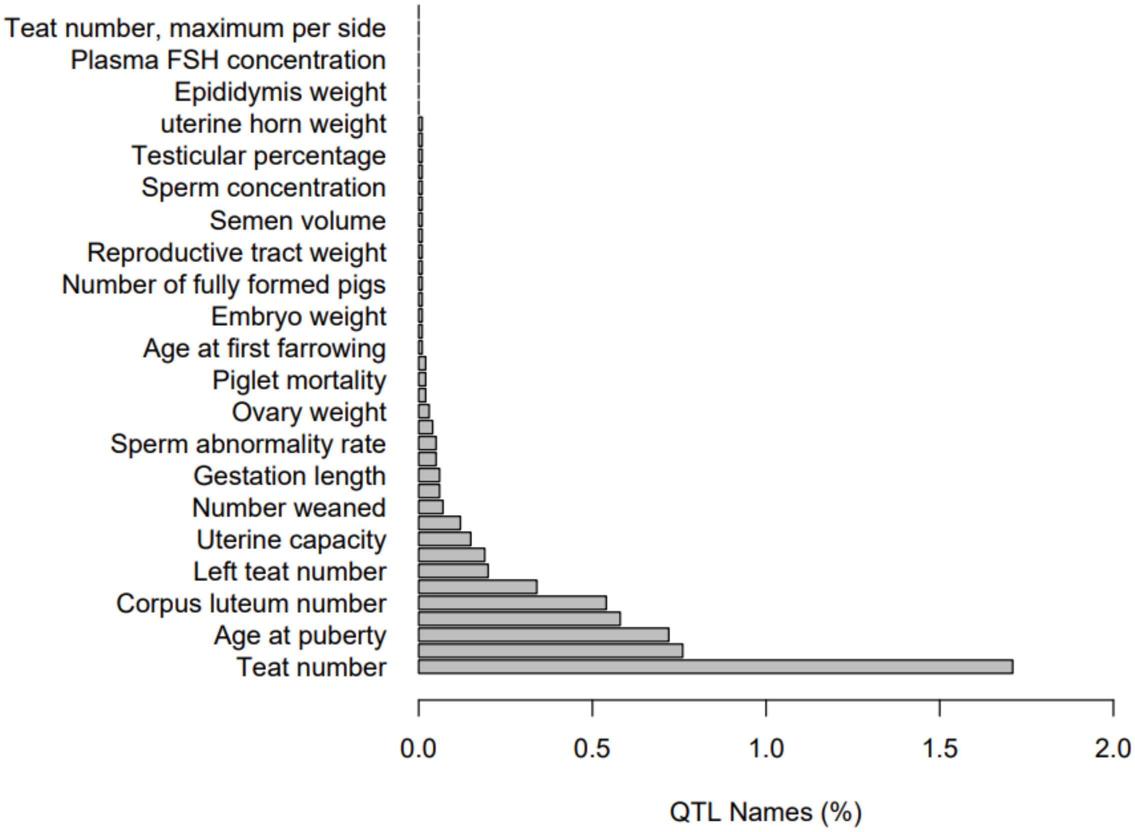


Figure S3. Percentage of reproduction class traits affected by CNVR

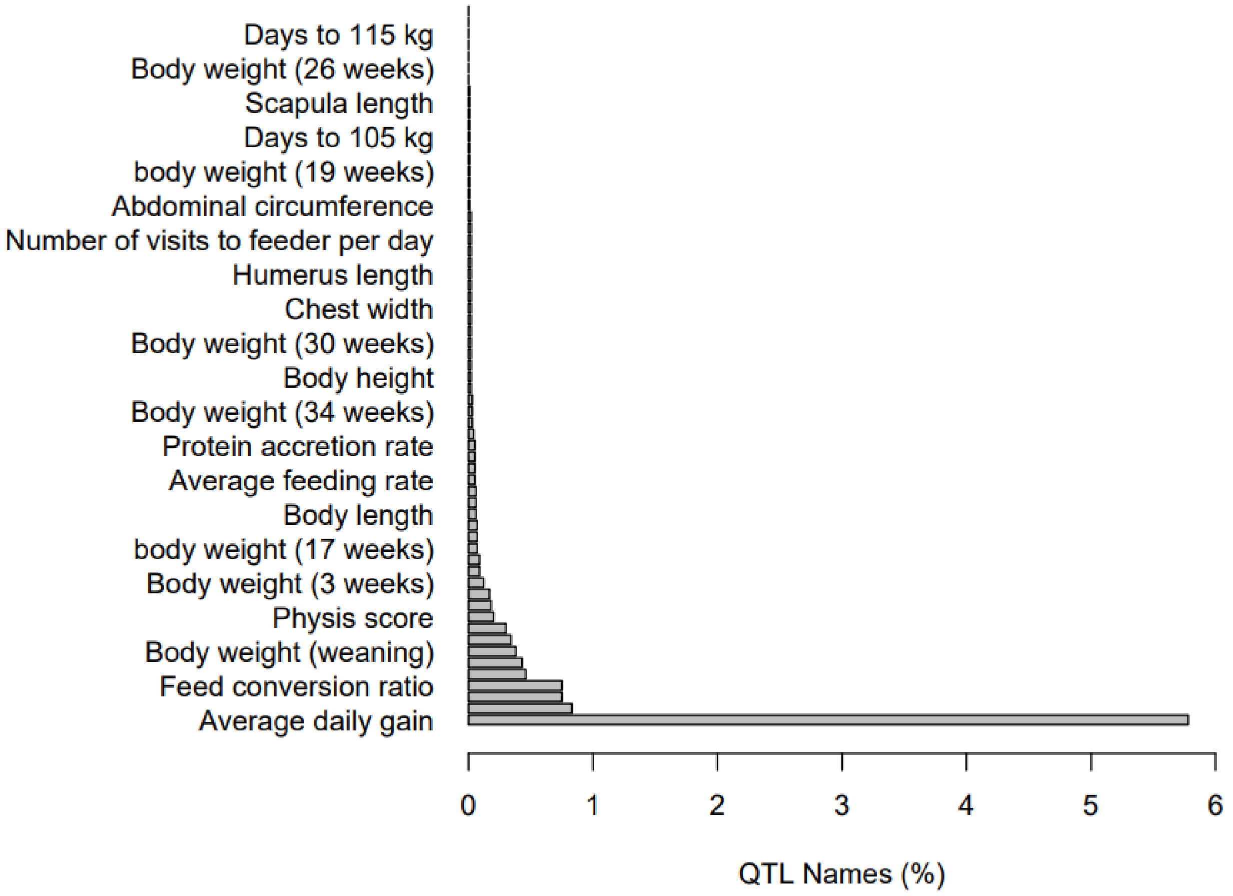


Figure S4. Percentage of production class traits affected by CNVR
